# Stool microbiome, pH and short/branched chain fatty acids in infants receiving extensively hydrolyzed formula, amino acid formula, or human milk through two months of age

**DOI:** 10.1186/s12866-020-01991-5

**Published:** 2020-11-09

**Authors:** Car Reen Kok, Bradford Brabec, Maciej Chichlowski, Cheryl L. Harris, Nancy Moore, Jennifer L. Wampler, Jon Vanderhoof, Devin Rose, Robert Hutkins

**Affiliations:** 1 Department of Food Science and Technology, University of Nebraska-Lincoln, Lincoln, NE 68588 USA; 2 Midwest Children’s Health Research Institute, LLC, 3262 Salt Creek Circle, Lincoln, NE 68504 USA; 3 Global Nutrition Science, Mead Johnson Nutrition, Evansville, IN 47721 USA; 4 Clinical Research, Department of Medical Affairs, Mead Johnson Nutrition, Evansville, IN 47721 USA; 5 Boston Children’s Hospital, Gastroenterology, 300 Longwood Avenue, Boston, MA 02115 USA; 6 Department of Food Science and Technology, University of Nebraska, 268 Food Innovation Center, Lincoln, NE 68588-6205 USA; 7 Department of Food Science and Technology, University of Nebraska, 258 Food Innovation Center, Lincoln, NE 68588-6205 USA

**Keywords:** Infant formula, Infant microbiota, *Bifidobacterium*, Short chain fatty acids

## Abstract

**Background:**

Early infant feeding with intact or extensively hydrolyzed (EH) proteins or free amino acids (AA) may differentially affect intestinal microbiota composition and immune reactivity. This multicenter, double-blind, controlled, parallel-group, pilot study compared stool microbiota from Baseline (1–7 days of age) up to 60 days of age in healthy term infants who received mother’s own milk (assigned to human milk [HM] reference group) (*n* = 25) or were randomized to receive one of two infant formulas: AA-based (AAF; *n* = 25) or EH cow’s milk protein (EHF; *n* = 28). Stool samples were collected (Baseline, Day 30, Day 60) and 16S rRNA genes were sequenced. Alpha (Shannon, Simpson, Chao1) and beta diversity (Bray Curtis) were analyzed. Relative taxonomic enrichment and fold changes were analyzed (Wilcoxon, DESEq2). Short/branched chain fatty acids (S/BCFA) were quantified by gas chromatography. Mean S/BCFA and pH were analyzed (repeated measures ANOVA).

**Results:**

At baseline, alpha diversity measures were similar among all groups; however, both study formula groups were significantly higher versus the HM group by Day 60. Significant group differences in beta diversity at Day 60 were also detected, and study formula groups were compositionally more similar compared to HM. The relative abundance of *Bifidobacterium* increased over time and was significantly enriched at Day 60 in the HM group. In contrast, a significant increase in members of Firmicutes for study formula groups were detected at Day 60 along with butyrate-producing species in the EHF group. Stool pH was significantly higher in the AAF group at Days 30 and 60. Butyrate increased significantly from Baseline to Day 60 in the EHF group and was significantly higher in study formula groups vs HM at Day 60. Propionate was also significantly higher for EHF and AAF at Day 30 and AAF at Day 60 vs HM. Total and individual BCFA were higher for AAF and EHF groups vs HM through Day 60.

**Conclusions:**

Distinct patterns of early neonatal microbiome, pH, and microbial metabolites were demonstrated for infants receiving mother’s own milk compared to AA-based or extensively hydrolyzed protein formula. Providing different sources of dietary protein early in life may influence gut microbiota and metabolites.

**Trial registration:**

ClinicalTrials.gov Identifier: NCT02500563. Registered July 28, 2015.

## Background

Cow’s milk allergy (CMA) is one of the most common food allergies affecting infants [1, 2]. Allergen exclusion, through use of formulas based on extensively hydrolyzed (EH) proteins, has traditionally been used to manage CMA. Amino acid (AA)-based formulas have also become available and may be used in more severe cases [3]. However, recent data suggest that use of AA-based formulas may delay the acquisition of immune tolerance [4], potentially due to the complete absence of allergenic epitopes that are required to induce oral tolerance through early exposure of intact dietary proteins [5, 6]. Unlike AA-based formulas, the presence of small peptide fragments in protein hydrolysates has been related to the stimulation of immune tolerance to cow’s milk protein [7]. This is particularly relevant with the advent of a new paradigm in the management of food allergy in infants that involves stimulation of the development of immune tolerance to food antigens. Whereas hydrolyzed formulas can minimize antigen contact, the mechanism of action also includes the induction of immune tolerance [8].

Oral tolerance development research has recently focused on the influence of host-microbe interactions on immune function, specifically that the presence of certain intestinal microbes could promote tolerance to dietary antigens [9–11]. For example, consumption of *Lactobacilllus rhamnosus* GG (in EH formula) by infants with CMA was reported to stimulate immune tolerance [4, 12, 13]. One hypothesized mechanism of enhanced immune tolerance is enrichment of butyrate-producing bacteria, with butyrate stimulating the development of regulatory T (T_reg_) cells. As known mediators of oral tolerance [14–17], T_reg_ cells have a demonstrated protective role, and a reduction could lead to an allergic phenotype [18, 19]. Ruohtula and colleagues demonstrated that an increase in highly activated T_reg_ cells was associated with colonization of butyrate-producing bacteria and suggested a very narrow window of opportunity (birth up to 3 months of age) for the primary prevention of atopic diseases [20]. Given the potential of protein hydrolysates to induce oral tolerance, we hypothesized that EH-based formulas encourage the production of butyrate through the enrichment of butyrate-producing microbes. Therefore, the primary goals of this study were to assess the effects of EH and AA-based formulas on the intestinal microbial composition of infants and the subsequent shift in formation of short and branched chain fatty acids. Preliminary findings were previously reported at the Nutrition 2019 - American Society of Nutrition annual conference [21, 22].

## Results

### Participants

**Fig. 1:**
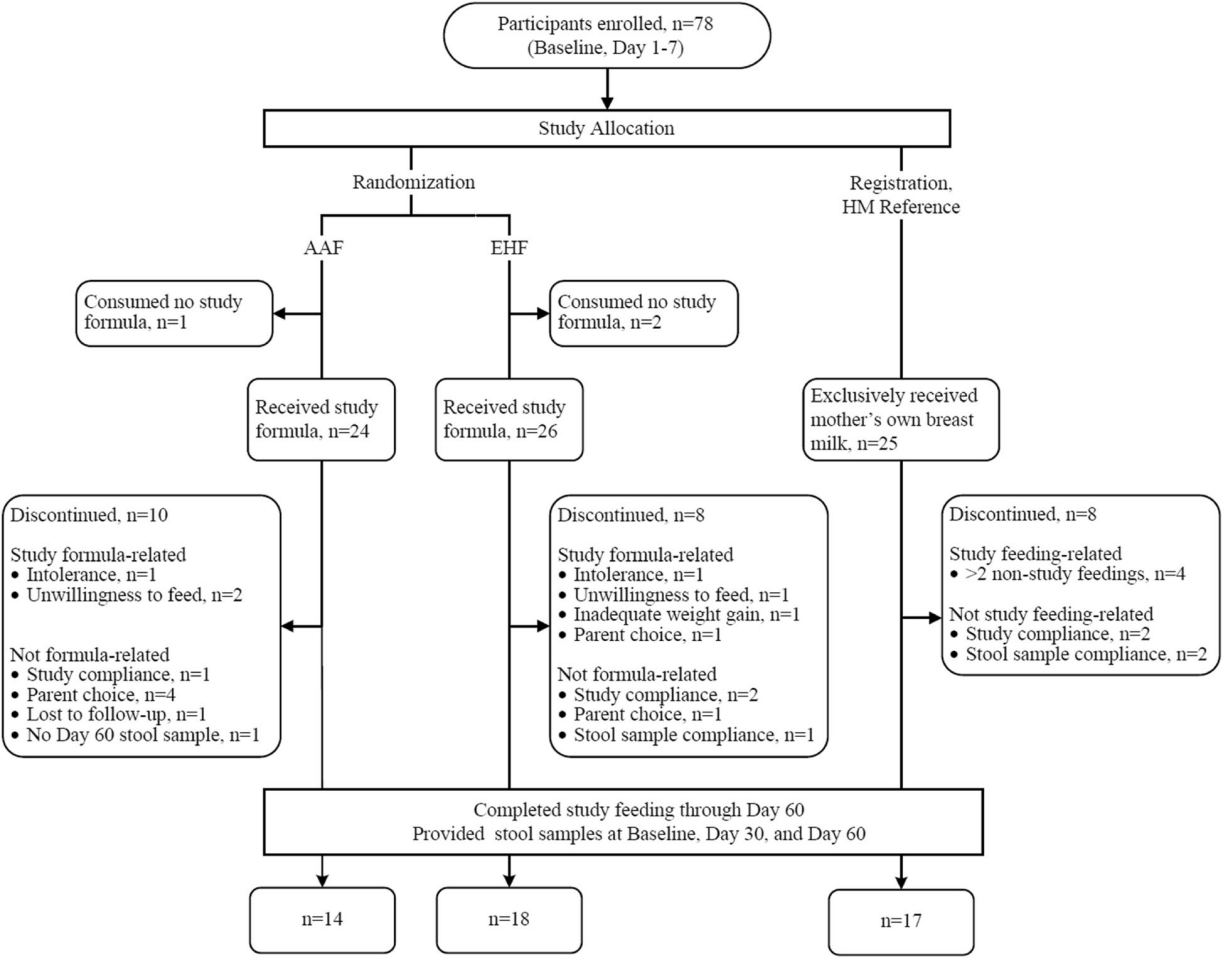
Timeline and participant flow diagram. **a** Timeline for the entire study duration. Fecal samples were collected based on specified days (infant’s age) as stated for each visit. Baseline samples were collected after meconium. **b** Flow chart describing subject exclusion and participation. Subjects who did not provide a sample or did not complete all 3 visits were excluded from the final analysis

A total of 78 participants were enrolled and assigned to a study group (EHF: *n* = 28; AAF: *n* = 25; HM: *n* = 25) (Fig. 1). Participants who were randomized but consumed no study formula (EHF: *n* = 2; AAF: *n* = 1) were not included in subsequent analyses. No differences in body weight, length, or head circumference were observed between groups at study enrollment (Table 1); sex, race and ethnic distribution were similar among groups (Additional File 1: Table S1). Statistically significant differences were detected at Day 30 in weight and length for the EHF vs HM group and at Day 60 in weight for the EHF and AAF vs HM groups. No differences in head circumference were detected at any study time point. No group differences in study formula intake (mean ± SE; fl oz./day) were detected between study formula groups at Day 30 (EHF: 27.8 ± 1.4 vs AAF: 25.8 ± 1.5: *P* = 0.355) or Day 60 (EHF: 33.4 ± 2.0 vs AAF: 28.7 ± 2.2: *P* = 0.128). No statistically significant group differences were detected for study discontinuation (EHF: *n* = 8, 31%; AAF: *n* = 10, 42%; HM: *n* = 8, 32%; *P* = 0.685) or discontinuation related to study formula (EHF: *n* = 4, 15%; AAF: *n* = 3, 13%). In the total study population, two participants discontinued due to formula intolerance as determined by the study investigator (EHF: *n* = 1; AAF: *n* = 1). Parental decision was the most common reason for discontinuation unrelated to study formula (5 participants). A total of four participants experienced serious adverse events and were categorized within the following body systems: Gastrointestinal (HM, *n* = 1), Respiratory (EHF, *n* = 1; AAF, *n* = 1; HM, *n* = 1), or Urogenital (EHF, *n* = 1). Participants who completed the study (EHF: *n* = 18; AAF: *n* = 14; HM: *n* = 17) provided stool data at all study time points and were included in subsequent analyses.

**Table 1:** Achieved weight, length, and head circumference^a^

Age (Days)	Group (n)	Weight (g)	Length (cm)	Head circumference (cm)
1–7 (Baseline)	AAF (24)	3294 ± 77	50.8 ± 0.04	34.3 ± 0.2
	EHF (26)	3296 ± 74	50.4 ± 0.04	34.9 ± 0.2
	HM (25)	3243 ± 76	50.9 ± 0.04	34.9 ± 0.2
30	AAF (16)	4073 ± 112	53.8 ± 0.05	37.2 ± 0.3
	EHF (20)	3912 ± 100^b^	52.8 ± 0.05^b^	36.9 ± 0.2
	HM (20)	4345 ± 100	54.8 ± 0.05	37.7 ± 0.2
60	AAF (15)	4939 ± 127^b^	57.0 ± 0.05	39.1 ± 0.3
	EHF (18)	4781 ± 118^b^	57.1 ± 0.05	38.9 ± 0.2
	HM (19)	5295 ± 114	58.2 ± 0.05	39.5 ± 0.2

^a^ Mean ± standard error (SE)

^b^ Significantly lower than HM, *P* < 0.05, two-tailed test

### Alpha and beta diversity

**Fig. 2:**
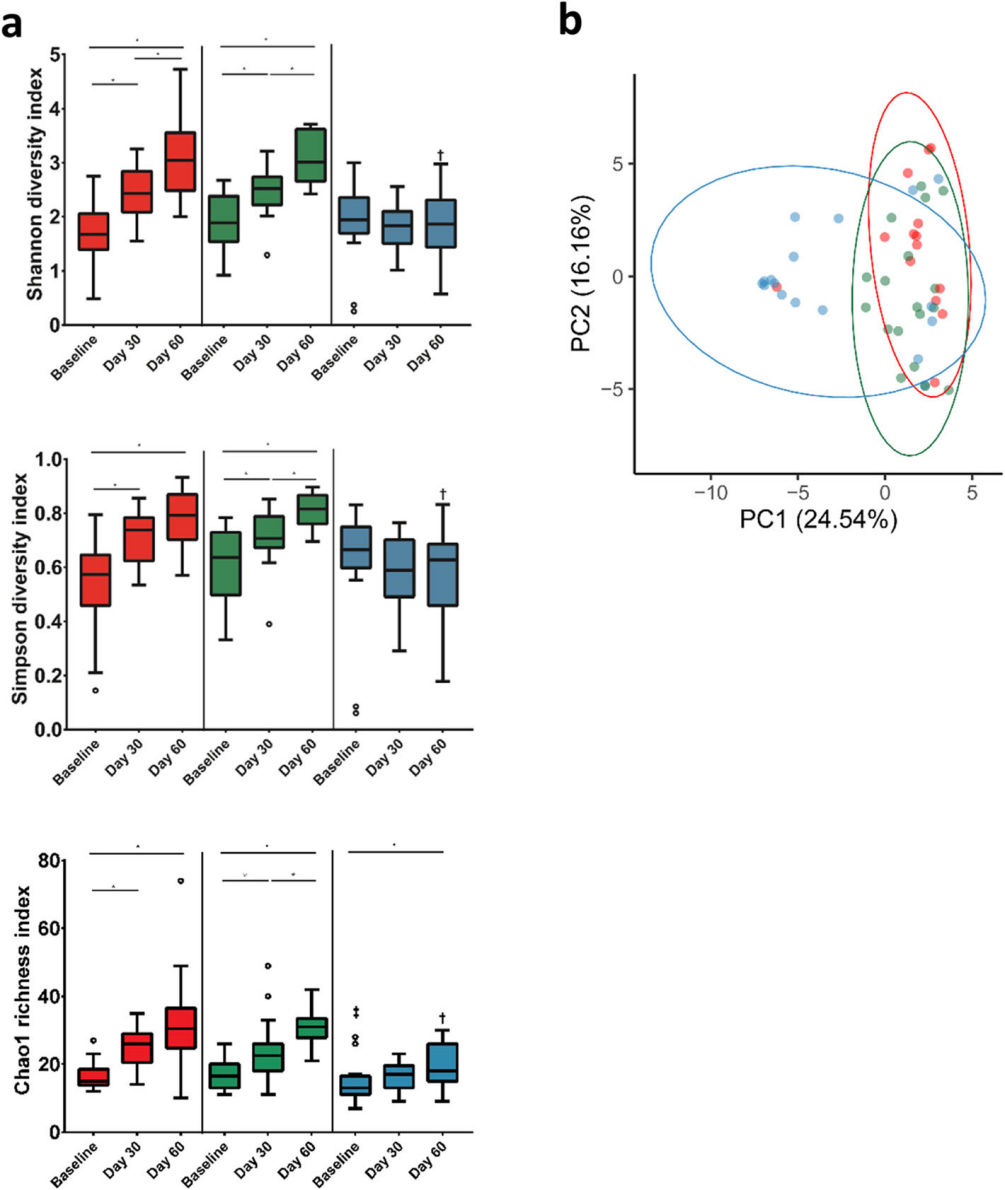
Alpha and beta diversity measurements of the infant microbiome across feeding groups. **a** Pairwise comparisons of alpha diversity indices (Shannon, Simpson, Chao1) were carried out between visits to examine specific comparisons of interest in each feeding group; amino acid (AAF; red), extensively hydrolyzed formula (EHF; green) and human milk (HM; blue). Boxplots show the median, first and third quartiles and the whiskers extend out to the 1.5 x IQR (Interquartile Range) of the upper and lower limit. * indicates significant difference between a pairwise comparison of group-visit combinations (FDR < 0.05). ‡ and † indicates that at baseline and day 60 respectively, the HM group was significantly different compared to the AA and EHF groups (FDR < 0.05). ○ represents outliers. **b** Comparison of community profiles at the ASV level between feeding groups at day 60. Principle coordinates analysis (PCoA) using Bray Curtis distance was carried out followed by PERMANOVA analysis (*p* = 0.001)

To compare overall changes in taxonomic profiles within and between each feeding group, alpha and beta diversity measures were calculated. Alpha diversity remained relatively stable from Baseline to Day 30 and from Baseline to Day 60 for the HM group when measured using Shannon and Simpson indices; whereas Chao1 richness significantly increased from Baseline to Day 60 (Fig. 2a). In contrast, alpha diversity increased over time in the AAF and EHF groups and was significantly higher for each compared to the HM group at Day 60 by all measures. Significant differences were also detected between Day 30 and Day 60 for the EHF (all measures) and AAF groups (Shannon index only). To assess community differences in beta diversity, PCoA plots were constructed using Bray-Curtis distance matrix (Fig. 2b). Overall community differences were detected (PERMANOVA, *P* = 0.001) with AAF and EHF communities displaying higher similarities at Day 60, compared to the HM group.

### Compositional change of genus level relative abundance between baseline and days 30 and 60

**Fig. 3:**
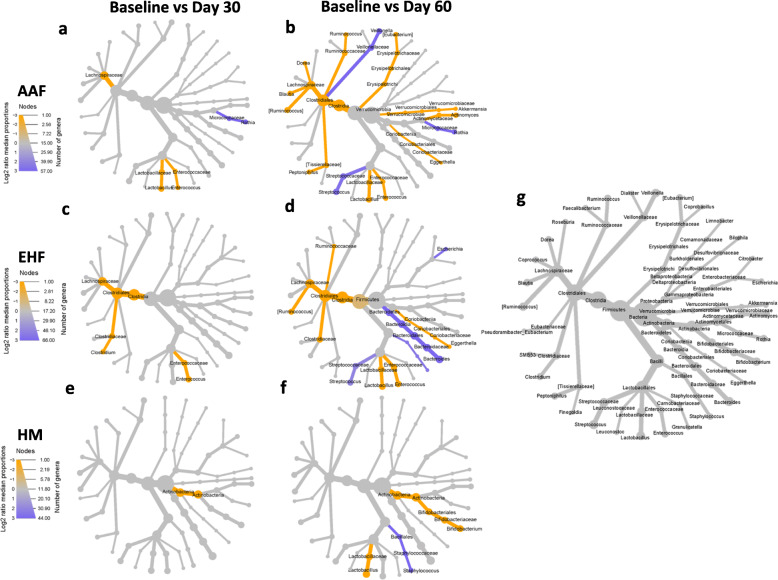
Heat trees comparing the relative abundances of taxa between **a**, **c**, **e** baseline and day 30 and **b**, **d**, **f** baseline and day 60 in the **a**. **b** amino acid (AAF)**, c**, **d** extensively hydrolysed formula (EHF) and **e,f** human milk (HM) groups. Taxa in orange are enriched at day 30 or day 60 while taxa in purple are enriched at baseline. Size of nodes correspond to the number of genera and color intensity corresponds to proportions. Taxonomic log2 fold change is calculated using median proportions. Only taxa that were significantly different between visits, as determined through Wilcoxon signed rank test (FDR < 0.05) are shown. Taxonomic tree in **g** acts as a reference and represents all genera present in the samples

Differences in taxonomic relative abundance between Baseline and Day 30 and between Baseline and Day 60 were evaluated using Wilcoxon tests and visualized for each group by heat tree mapping. For all 3 groups, significant taxonomic changes began to appear by Day 30 (Fig. 3). In the AAF and EHF groups, members of Firmicutes (e.g. *Lachnospiraceae*) were already significantly higher at Day 30 (Fig. 3a,c). In the AAF group, there was an observed increase in *Lactobacillus* and *Enterococcus* and a decrease in *Rothia* at both Days 30 and 60 (Fig. 3a,b). At Day 60, an increase was observed in *Akkermansia*, *Actinomyces*, *Eggerthella*, *Peptoniphilus*, *[Ruminococcus], Blautia, Dorea, Ruminococcus and [Eubacterium]* together with a decrease in *Veilonella* and *Streptococcus*. In the EHF group, there was an observed increase in *Enterococcus* at both Days 30 and 60 (Fig. 3c,d). By Day 60, *Eggerthella*, *Lactobacillus, [Ruminococcus]* and members of *Clostridiaceae* and *Ruminococcaceae* were significantly higher while *Escherichia*, *Bacteroides* and *Streptococcus* had significantly decreased. For the HM group (Fig. 3e,f), members of Actinobacteria were significantly higher at Day 30. By Day 60, a significant increase in *Bifidobacterium* and *Lactobacillus* and a significant decrease in *Staphylococcus* were observed.

**Fig. 4:**
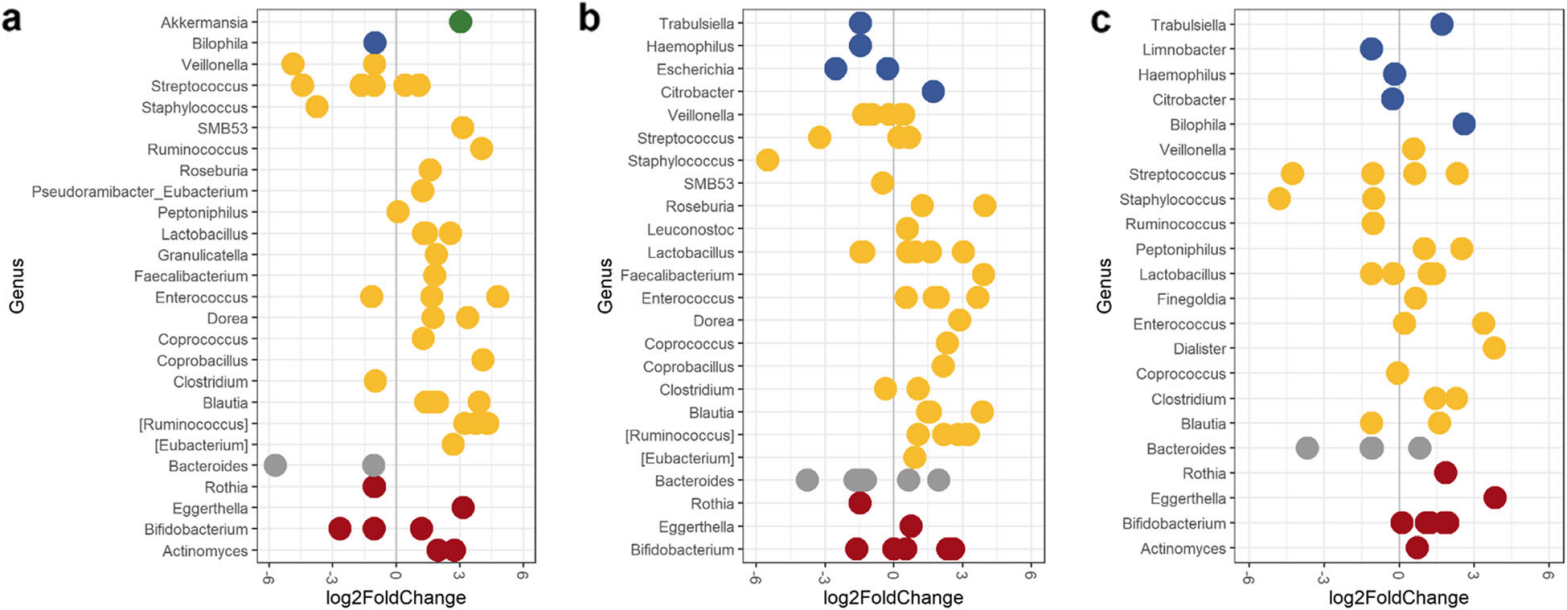
DESeq2 analysis of differentially abundant ASVs between baseline and day 60 for each feeding group; the **a** amino acid (AAF), **b** extensively hydrolysed formula (EHF) and **c** human milk (HM) groups. Shrinkage estimations of log2 fold change values for each ASV were computed and each point represents an ASV that was significantly different (FDR < 0.05). Each color represents a phylum; Actinobacteria (red), Bacteroidetes (grey), Firmicutes (yellow), Proteobacteria (blue) and Verrucomicrobia (green)

To further evaluate the differential abundance of specific Amplicon Sequence Variants (ASVs, unique DNA sequence from a marker gene) from Baseline and Day 60, the DESeq2: differential gene expression analysis based on the negative binomial distribution package in R was used to compute log 2-fold changes (log_2_FC) of ASVs. Similar results were observed as before. In the AAF (Fig. 4a) and EHF (Fig. 4b) groups, a significant shift in members of Firmicutes were observed. This included an increase in *Enterococcus* and *Blautia* species and species belonging to the Clostridia IV and XIVa clusters, such as *Ruminococcus*, *Dorea*, *Eubacterium*, *Roseburia*, *Faecalibacterium*, and *Coprococcus*. A significant decrease in species of *Staphylococcus*, *Streptococcus*, *Veillonella,* in addition to other decreases in *Bacteroides* and *Rothia,* were also observed. In the HM group (Fig. 4c), overall shifts in members of Firmicutes were less apparent. However, a significant increase in *Dialister* and *Finegoldia*, which was not observed in the formula-fed groups occurred in the HM group.

When organisms associated with butyrate production were assessed, two different *Roseburia* ASVs (log_2_FC of 1.2 and 3.9), and a *Faecalibacterium prausnitzii* ASV (log2FC of 3.9) increased in the EHF group. *Roseburia* and *Faecalibacterium* were both predictors in the random forest variable importance plots for the EHF group (Additional File 1: Fig. S1b). In comparison, a *Roseburia* ASV and a *Faecalibacterium prausnitzii* ASV increased in the AAF (log_2_FC of and 1.6 and 1.8 respectively) group. A significant enrichment of *Akkermansia* (log_2_FC of 3.06) was observed in the AAF group (similarly observed by Wilcoxon test and random forest analysis) (Fig. 3a, 4a, Additional File 1: Fig. S1a). Changes in different bifidobacteria species occurred in each group. A *Bifidobacterium longum* ASV was enriched in the HM group (log_2_FC of 2) but decreased in the AAF (log_2_FC of − 2.7) and EHF (log_2_FC of − 1.6) groups. *Bifidobacterium pseudocatenulatum* corresponded to a single ASV enriched in the AAF group (log_2_FC of 1.2) and the two most enriched *Bifidobacterium* ASVs in the EHF group (log_2_FC of 2.4 and 2.7). Random forest analysis also demonstrated that *Bifidobacterium* ASVs were the main predictors reflecting whole microbiome changes between Baseline and Day 60 for the EHF and HM groups (Additional File 1: Fig. S1).

### Group differences in genus level relative abundance at day 60

**Fig. 5:**
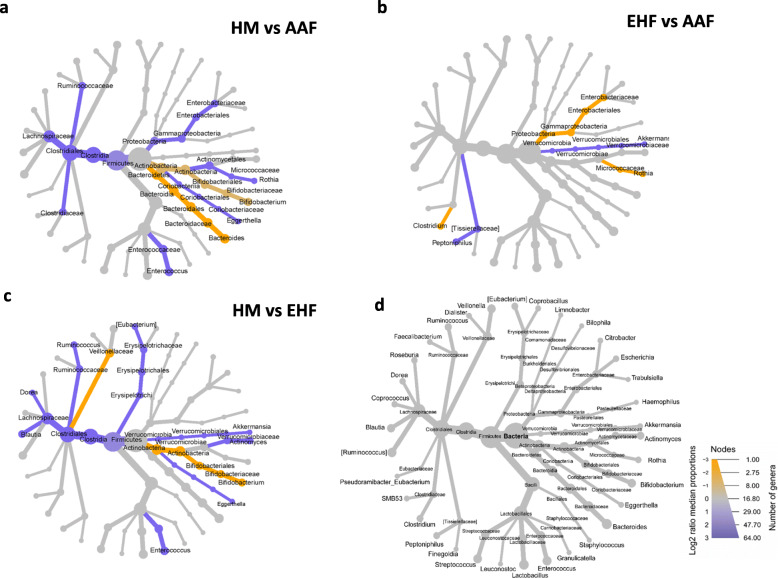
Differential heat trees based on pairwise comparisons of taxonomic relative abundance between feeding groups at day 60; **a** human milk (HM; yellow) vs amino acid (AA; purple), **b** extensively hydrolyzed formula (EHF; yellow) vs amino acid (AAF; purple) and **c** human milk (HM; yellow) vs extensively hydrolyzed formula (EHF; purple). Taxonomic tree in **d** acts as a reference and represents all genera present in the day 60 samples. Taxonomic log2 fold change is calculated using median proportions. All colored taxa are significantly different between feeding groups (Wilcoxon rank sum test; FDR *p* < 0.05). Size of nodes correspond to the number of genera and color intensity corresponds to proportions

Relative abundance between study groups at Day 60 was also analyzed by a pairwise Wilcoxon comparison (Fig. 5). *Bifidobacterium* was significantly higher and Firmicutes significantly lower for the HM group compared to AAF (Fig. 5a) and EHF (Fig. 5c) groups. Enterobacteriaceae, *Rothia* and *Clostridium* were significantly higher and *Akkermansia* and *Peptoniphilus* were significantly lower for the EHF compared to the AAF group (Fig. 5b).

### pH and S/BCFA

**Fig. 6:**
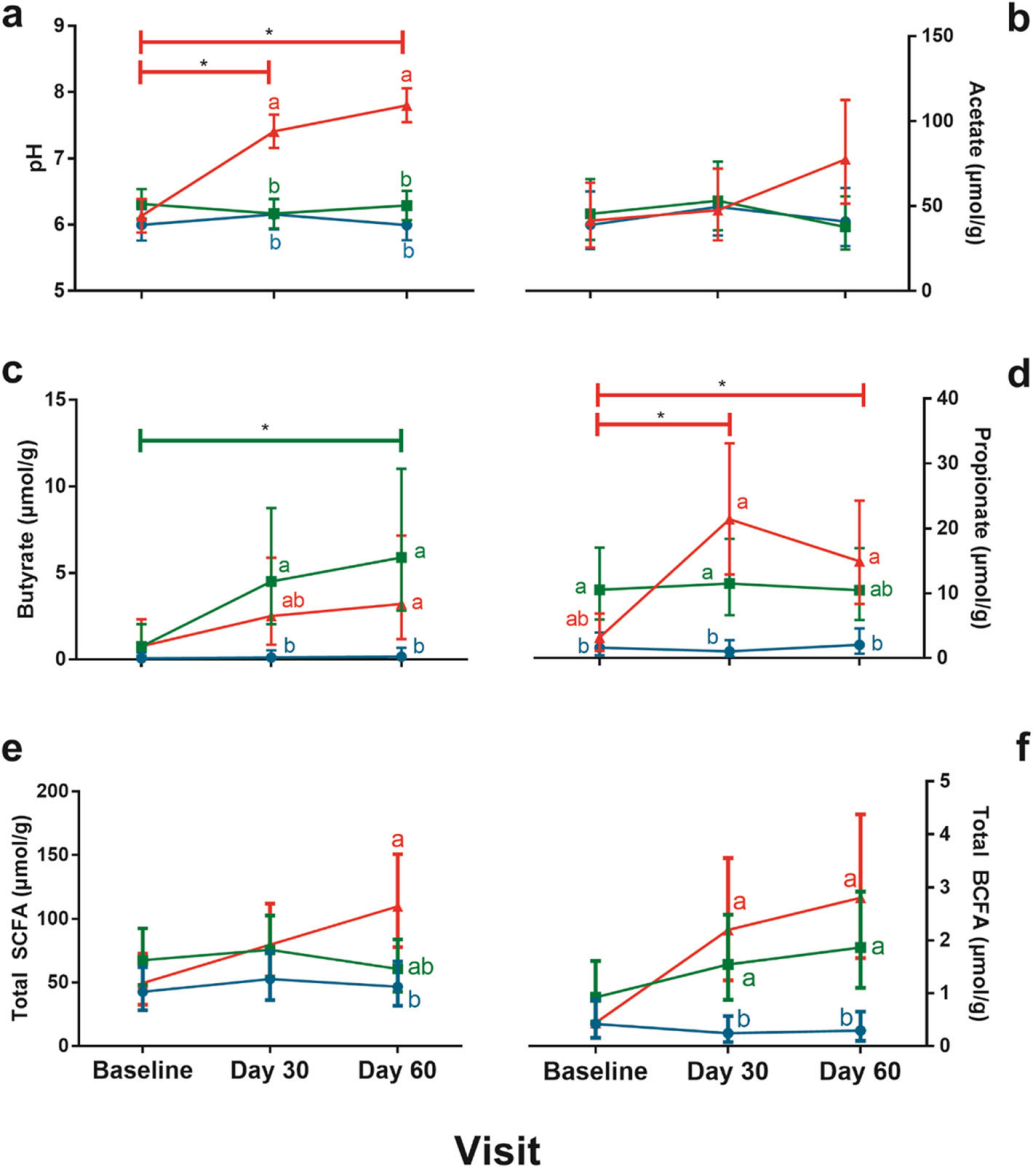
pH and S/BCFA analysis of stool samples. For the different feeding groups; amino acid (AAF; red), extensively hydrolyzed formula (EHF; green) and human milk (HM; blue), **a** pH, **b** acetate, **c** butyrate, **d** propionate, **e** total SCFA and **f** total BCFA were measured at each visit. A repeated measure analysis of variance (ANOVA) using a Toeplitz covariance structure was used with Bonferroni’s post-hoc test. Each point represents the mean and error bars represent upper and lower limits at a 95% confidence interval. * represents significant difference between visits for AAF in **a** pH and **d** propionate and EHF in **c** butyrate (*p* < 0.05). Points that do not share the same letter(s) at a given visit are significantly different from each other (*p* < 0.05)

Stool pH values (mean ± SE) were similar among groups at Baseline with no significant changes for EHF and HM through Day 60. However, compared to EHF and HM, mean pH values in the AAF group were significantly higher at Days 30 (*P* < 0.01 for both) and 60 (*P* < 0.01 for both) (Fig. 6a). With the exception of propionate (HM significantly lower vs EHF group), no significant group differences in S/BCFA (μmol/g; mean ± SE) were detected at Baseline. Butyrate increased significantly from Baseline to Day 60 in the EHF group (*P* = 0.026) and was significantly higher vs HM at Days 30 and 60 (*P* < 0.01) (Fig. 6c). Butyrate was also significantly higher for AAF vs HM at Day 60 (*P* = 0.038). In the AAF group, propionate levels significantly increased from Baseline to Day 30 (*P* < 0.01) and from Baseline to Day 60 (*P* = 0.026) and was also significantly higher compared to the HM group at Days 30 (*P* < 0.01) and 60 (*P* = 0.01) (Fig. 6d). Total SCFA was similar for all groups through Day 30 but was significantly higher in the AAF versus HM group at Day 60 (*P* = 0.04) (Fig. 6e). Both the AAF and EHF groups displayed similar trends of total BCFA production and were significantly higher compared to HM at Day 30 (*P* < 0.01, *P* = 0.015 respectively) and Day 60 (*P* < 0.01 for both) (Fig. 6f, Additional File 1: Fig. S2). Spearman correlations were calculated to associate taxa at the genus level with individual metabolites (Fig. S3). Notable correlations include positive correlations between acetate and *Bifidobacterium* and between butyrate and members of the Clostridia IV and XIVa clusters (*Roseburia*, *Faecalibacterium*, *Dorea*, *Coprococcus*, *Clostridium* and *[Ruminococcus]*). Butyrate was also positively correlated with *Leuconostoc*, *Pseudoramibacter_Eubacterium*, *Enteroccocus*, *Eggerthella*, *Blautia*, *Akkermansia*, *[Eubacterium]* and *Citrobacter*.

## Discussion

One of the most important factors affecting the development and composition of the infant gut microbiome is nutrition, specifically, the type of infant feeding [23–25]. In particular, human milk and infant formulas have been previously demonstrated to induce differences in microbiota composition [26, 27]. In the current study, two hypoallergenic infant formulas that had different sources of protein were compared to human milk in infants from birth to Day 60. These formulas have been previously or are currently marketed, and have been demonstrated to adequately support typical growth and safety [28]. In this study, group differences in achieved growth between study formula groups and the HM reference group were detected, as could be expected. Weight gain in healthy breastfed infants has been reported as typically faster compared to those receiving formula feeding in the first few months of life, but slower over for the remainder of infancy [29].

The major groups of bacteria detected among all study groups and time points belonged to phyla known to be associated with the infant gut microbiome, including Firmicutes, Actinobacteria, Proteobacteria and Bacteroidetes [30, 31]. In particular, enrichment of *Bifidobacteriaceae* in the HM reference group was consistent with previous gut microbiome characteristics of infants receiving mother’s own milk [13, 32]. In general, *Bifidobacterium* spp. typically dominate the infant gut early in life [33] especially *B. bifidum*, *B. breve* and *B. longum,* but slowly attenuate during adolescence and adulthood [34, 35]. Human milk serves as an initial source of *Bifidobacterium* [36, 37] and, importantly, also provides a continuing source of human milk oligosaccharides (HMOs) throughout lactation. HMOs are specifically metabolized by groups of bifidobacteria, *B. longum* in particular [38–41].

Abundance of bifidobacteria in the infant gut has been suggested as protective against development of allergic diseases [20, 42–46], and may be an advantage associated with breastfeeding [47]. We observed that abundance of a *B. longum* ASV increased in the HM reference group but decreased in both study formula groups. In contrast, a significant increase in *B. pseudocatenulatum* was demonstrated in both study formula groups, particularly the EHF group. The presence of *B. pseudocatenulatum* has previously been observed in both breastfed infants and those receiving infant formula [48, 49], although the role of this species in the development of immune or other health parameters is unclear [48, 50].

In the current study, the trend toward higher alpha diversity was similar for infants receiving EHF or AA-based formulas, suggesting the compositional evenness and richness of taxa in the infant gut was influenced by feeding during early life. Similarly, stool microbiota diversity has been demonstrated to be significantly higher in infants receiving formula vs human milk in US [51] and Korean populations [52]. In the US study, lower diversity in infants receiving human milk was likely due to the dominance of *Bifidobacterium*, which are enriched by HMOs [35, 38, 53]. In the current study, diversity within the HM group was stable through Days 30 and 60 (albeit an increase in Chao1 richness at Day 60), reflecting minimum change in gut community composition throughout the study period. The increase in Chao1 richness was likely influenced by an increase in the number of rare species. In contrast, the increased diversity over time for infants receiving EH or AA-based formulas was associated with enrichment of members of the phylum Firmicutes.

Whereas beta diversity measures demonstrated a lack of defined separation between groups, the overlap between the AAF and EHF groups at Day 60 further suggested greater similarity in community composition compared to the HM reference group. Nevertheless, the association between diversity and health remains undefined, partly because diversity is not reflective of ecosystem function and stability [54, 55]. Instead, the underlying ecological mechanisms driven by functionally conserved species should be considered in addition to community diversity. Although the gut microbiome is more diverse in infants receiving formula compared to human milk, the association between atopy and microbial diversity in the infant gut (as assessed across several studies) remains inconclusive [56–60]. For example, children with food sensitization in early life had lower alpha diversity compared with children without these conditions [61].

Downstream taxonomic analyses yielded similar results, suggesting that different sources of dietary protein may have been responsible for these changes in gut community composition. These changes may further be associated with the differential production of metabolites such as S/BCFA, which are known to communicate with the immune system [62].

The enrichment of Firmicutes in both study formula groups was consistent with previous reports and reflected a more adult-like microbiome [52, 63, 64]. The phylum Firmicutes includes commensal *Clostridia* species and related taxa known to produce butyrate and induce T_reg_ cells in germ-free mice [16, 65]. Indeed, a majority of the ASVs associated with butyrate production identified in the formula groups belonged to Clostridia IV and XIVa clusters (including *Ruminococcus*, *Dorea*, *Lachnospira*, *Eubacterium*, *Roseburia*, *Faecalibacterium* and *Coprococcus* [66, 67]), potentially accounting for the observed increase in butyrate in these groups. This is supported by the positive correlations observed between butyrate concentrations and these specific taxa. Furthermore, ASVs corresponding to *Faecalibacterium* and *Roseburia* identified in the EHF group (by both DESeq2 differential abundance and random forest analysis) had as much as a log_2_ fold increase of 3.9. Butyrate production through cross-feeding reactions between the observed bifidobacteria and butyrate-producing organisms (similar to species demonstrated in the EHF group) has also been demonstrated previously [68, 69]. Butyrate is the main energy source for colonic epithelial cells and was shown to regulate gut permeabilty [70]. Butyrate producers *Roseburia* and *F. prausnitzii* are found in high numbers in the gut of healthy adults [66, 71, 72] and *F. prausnitzii* has been studied as a beneficial commensal correlated with improved health in adults [71, 73]. *Faecalibacterium* was also found to be lower in abundance in children belonging to two different birth cohorts who had higher risks in the development of atopy [74] and in children that developed allergic diseases [20]. Interestingly, enrichment of *A. muciniphila* was only observed in the AAF group with a significant fold increase from baseline to Day 60 along with higher levels of propionate throughout. *A. muciniphila* is pH sensitive, has optimum growth in environments closer to neutral pH, and produces propionate as a metabolic end product of mucin degradation [75]. Some of the described benefits of propionate include the improvement of glucose metabolism in adults [76, 77] and the prevention of allergic diseases in children [78]. An association between longer uninterrupted sleep and an increase in propionate was also observed in infants of the Baby-Led Introduction to SolidS (BLISS) cohort [79].

Distinct patterns of pH and microbial metabolites were also observed for infants receiving EH or AA-based formulas compared to mother’s own milk. Although higher stool pH has been previously observed in a small study of infants receiving formula vs human milk [80], a dramatic shift over the past century in mean fecal pH values among breastfed infants in developed countries (an increase from 5.0 to 6.5) was recently reported [81]. The researchers suggested that this shift was correlated with the presence and absence of specific taxa; specifically, increased pH values were associated with an increase in dysbiosis-associated taxa (*Clostridiaceae*, *Enterobacteriaceae*, *Peptostreptococcocaceae* and *Veillonellaceae*) and a decrease in *Bifidobacteriaceae*. In the results presented here, the mean fecal pH of the AAF group at Days 30 and 60 was more than 1 pH unit higher than the range reported in Henrick et al. [81] and significantly higher compared to the EHF and HM reference groups. Similar taxonomic shifts were also observed, including a significant increase in *Clostridiaceae* and *Enterobacteriaceae* and decrease in *Bifidobacteriaceae* in the AAF group compared to the HM group. The role of these species in amino acid fermentation and metabolism can potentially influence the observed differences in pH [82, 83]. Additionally, increased BCFA in the AAF and EHF groups was consistent with free branched-chain amino acid fermentation in the colon [84, 85].

Although butyrate was higher for both study groups compared to HM at Day 60, a significant increase from baseline to Day 60 was evident only for the EHF group. This finding, along with the presence of butyrate-producing organisms in the EHF group, may provide insights into the T_reg_-associated mechanisms by which an EH formula more effectively accelerates oral tolerance acquisition in children with CMA versus an AA-based formula [4, 20, 86]. Previously, the role of FOXP3^+^ T_reg_ cells and its mechanisms in the development of oral tolerance have been described [86]. Other studies have demonstrated the presence of higher frequencies of circulating T_reg_ cells in children who acquired spontaneous tolerance [18] and in children who outgrew CMA [87]. Importantly, butyrate has been linked to T_reg_ stimulation by increasing acetylation of FOXP3 and improving accessibility of transcriptional regulators at the gene promoter region and other enhancer elements [16]. The association between butyrate-producing bacteria and T_reg_ cell maturation was also evident after the first year of life in a birth-cohort of Estonian and Finnish children [20]. In that study, highly activated T_reg_ cells increased and demonstrated a strong association with the butyrate-producing bacteria, thus increasing diversity of the intestinal microbiome. Furthermore, shotgun metagenomic sequencing of infant stool samples from the Canadian Healthy Infant Longitudinal Development (CHILD) cohort demonstrated that infants who developed allergic sensitization later on in life were deficient in butyrate-producing genes since 3 months of age [58].

Early diet choices may influence childhood development during the first 1000 days of life and beyond. Together with previous reports, our study suggests that using an EH formula may accelerate oral tolerance acquisition by supporting a microbiome that is capable of producing butyrate. The EH formula was associated with lower pH, shifted the microbiota composition toward butyrate production (e.g., *Roseburia*, *F. prausnitzii*), and altered the SCFA profile (more butyrate vs propionate and acetate) compared to an AA-based formula. Further research is warranted in this area.

## Conclusions

The gut microbiome plays a critical role in the regulation of allergic immune responses. Several distinct patterns of early neonatal microbiome, pH, and microbial metabolites were demonstrated for infants receiving mother’s own milk compared to amino acid-based or extensively hydrolyzed protein formulas. The abundance of *Bifidobacterium* was higher in breastfed infants, and Firmicutes were dominant in both of the infant formula groups. Shifts in specific bacteria species were associated with production of metabolites, particularly butyrate, that is known to communicate with the host immune system. Within the group receiving an EH formula, specific taxonomic shifts included the enrichment of butyrate producers, potentially linked to the induction of oral tolerance to cow milk proteins. However, it is not clear if these outcomes are mediated through changes in the microbiome.

Results from this study suggest that formulas with different protein sources affect the microbiome differently, possibly impacting development and functionality of the immune response. Longitudinal studies that include both microbiome measurements and observations of immunological effects are needed to fully understand the mechanisms by which these infant formulas function. In addition, use of whole shotgun metagenomic sequencing to uncover species and microbial pathways that contribute towards the production of beneficial metabolites is needed. Finally, although these findings using stool samples provide a basis for understanding the influence of particular types of formula feeding on the infant colonic microbiota, just as for other studies based on fecal samples, they may not fully reflect the conditions along the entire intestinal tract.

## Methods

### Study population

Healthy, term infants (1 to 7 days of age) were recruited at four clinical sites in the United States. Eligible infants were singleton births at 37–42 weeks gestational age (GA) with appropriate birth weight for GA (defined as birth weight between and inclusive of 5th and 95th percentiles [88, 89]; and started either infant formula feeding or mother’s own breast milk feeding within 24 h of birth. Infants whose mothers had chosen not to breastfeed were solely formula-fed at least 24 h prior to randomization. Infants from mother who intended to breastfeed exclusively received mother’s-own breast milk from Day 1 (day of birth was considered Day 0) through study registration. Exclusion criteria included: caesarean delivery; birth mother who had Type 1 diabetes; history of underlying disease or congenital malformation likely to interfere with normal growth and development or participant evaluation; evidence of feeding difficulties (from breast or bottle) or history of formula intolerance; and immunodeficiency; signs of acute infection or use of antibiotics (including topical antibiotics in the diaper area) at study randomization/registration; and planned use of probiotics during the study period.

### Study design

This study was designed to examine the impact of early feeding of EH casein formula on intestinal microbiota composition. At the time of study inception, little data existed on which to base a formal sample size calculation. However, microbiota differences had been demonstrated in a study with two groups of 18 infants each [50]. Therefore, 20 infants per group were targeted to complete the study feeding period. In this double-blind, randomized, controlled, parallel-designed, prospective trial participants were enrolled between August 2015 and May 2017. Parents or legally authorized representative(s) provided written informed consent prior to enrollment. The research protocol and informed consent forms observing the Declaration of Helsinki were approved by Schulman IRB. Participants receiving infant formula were randomly assigned to receive one of two study formulas (Mead Johnson Nutrition, Evansville, IN): an amino acid-based infant formula (AAF; marketed PurAmino) or an extensively hydrolyzed protein infant formula (EHF; previously marketed Nutramigen®) through 60 days of age. Neither study formula included added *Lactobacillus rhamnosus* GG. Similarly to Yeiser at al. ([90]), a computer-generated randomization schedule was prepared and inserted into sealed envelopes that were consecutively-numbered. The individual study formulas were designated using one of two unique codes. Study formulas assigned to each participant were determined after opening the next sequential envelope. The codes were known only to the sponsor. Formulas were provided directly to parents at each visit prior to completion of the study or to withdrawal.

Neither the sealed envelopes nor the product labels permitted unblinding by the study site. In addition, the personnel involved in monitoring the study were blinded to product identification. Only in the event of a medical emergency that required knowledge of the study formula for managing the participant’s management, could blinding be broken. In this study, there were no instances where it was necessary to prematurely break the study code.

Participants exclusively receiving mother’s own breast milk were registered and assigned to a human milk (HM) reference group. Study visits occurred at Baseline (1–7 days of age), 30 (±3) days and 60 (±3) days of age. Anthropometric measures (body weight, length, and head circumference) were recorded at all study visits. Parents collected Baseline stool samples after meconium passed and ≤ 24 h of participant study enrollment. Day 30 and 60 stool samples were collected ±24 h of the study visit and compliant samples were stored onsite at − 20 °C. Study completion was defined as participants who provided a protocol-compliant stool sample at all three study time points. (Stool sample compliance criteria: Additional File 1, Table S2). Serious adverse events were collected throughout the study.

### pH and S/BCFA analysis

A standard pH meter (Orion Research Inc., Boston, Mass., USA) with a micro combination electrode (Fisher Scientific, Lenexa, Kansas) was used to measure pH of diluted (1:10, deionized water) and homogenized stool samples. Short chain fatty acids (SCFA; acetate, butyrate and propionate) and branched chain fatty acids (BCFA; isovalerate and isobutyrate) were measured using gas chromatography as described previously [91] with slight modifications. Briefly, diluted, homogenized stool samples (1:10; phosphate-buffered saline) were centrifuged (8000×*g* × 5 min); pellets were stored at − 20 *°*C (for subsequent DNA extraction). Supernatant was collected (0.4 ml), and diethyl ether extracts were prepared and quantified by gas chromatography (Clarus 580; PerkinElmer, Waltham, MA, USA) using a fused silica capillary column (Nukol 30 m × 0.25 mm id × 0.25 μm film; Sigma-Aldrich, St. Louis, MO, USA) and a flame ionization detector. Quantification of S/BCFA was based on calculating response factors relative to known concentrations of 2-ethyl butyric acid. Internal standards of 2-ethyl butyric were used during quantification to correct for any variability caused by loss of analyte.

### DNA extraction, 16S rRNA community sequencing and analysis

DNA from stool samples were extracted using phenol-chloroform as described by [92] with the exception that all incubation times were carried out for 30 min and DNA was recovered with 100 μl of DNase-free water. Amplicon sequencing was performed by Neogen GeneSeek Operations (Lincoln, NE). Briefly, 2x250bp paired-end 16S rRNA regions of the DNA samples were sequenced with an Illumina MiSeq sequencer. Primers used were 515F (5′-GTGCCAGCMGCCGCGGTAA-3′) and 806R (5′-GGACTACHVGGGTWTCTAAT-3′), flanking the 515 and 806 region. Barcodes were attached to the 806R primers. A total of 8,789,037 sequences were obtained with a mean of 51,099 sequences per sample.

Sequence analysis was conducted using QIIME 2.0: Quantitative Insights Into Microbial Ecology [93]. Paired-end raw sequences were de-multiplexed and imported into QIIME. FastQC was used to check for per sample sequence quality and DADA2 was used to remove chimeric sequences [94]. To retain high quality sequences, forward reads were truncated to 240 bp and reverse reads were truncated to 200 bp. In total, 6,043,211 sequences were de-replicated into unique amplicon sequence variants (ASV) and a list of representative sequences with 619 features was created. Taxonomy was successfully assigned to 557 features using the Greengenes database with a pre-trained classifier based on 99% sequence identity. In the Greengenes database,square brackets around taxonomy indicate suggested but not verified taxonomy by the database curators. Alpha and beta diversity measures were computed using a sample depth of 9351 sequences. Species-level taxonomic information that could not be obtained from the Greengenes database was further identified through BLASTn against the NCBI Refseq database and the top hits were used as an identifier.

### Statistical analysis

Analyses on measured pH and S/BCFA were performed using Statistical Analytical System (SAS) software ver. 9.4 (SAS Inst., Cary, NC, USA). Differences in pH and S/BCFA production between each group-visit combination were analysed with a repeated measure analysis of variance (ANOVA) using a Toeplitz covariance structure. The differences between the least square means of fixed effects were used to compare between group-visit combinations. To account for multiple comparisons, a Bonferroni adjustment was used and *p* < 0.05 was considered significant. In this model, ‘group’ and ‘visit’ were the fixed effects while ‘visit’ was the repeated measure and subject within group was used as the random error. Data transformations were used to adjust for normality; double square root transformation for acetate, butyrate and total SCFAs and cubic transformation for propionate and isovalerate. To adjust for the inflated number of zeros observed (not detectable) in the measurement of isobutyrate throughout the samples, an analysis of probability of a zero observation was carried out. Then, a modified log transformation was used to adjust for normality after the removal of zeros from the dataset.

Statistical analysis for 16S rRNA community sequencing was done in QIIME2 and R (ver 3.6.1). Shannon, Simpson, and Chao1 richness indices were computed at the ASV level to measure alpha diversity. For every group-visit combination, pairwise comparisons of interests (between visits in all three feeding groups and between groups at visits 1 and 3) were made using Kruskal-Wallis tests. FDR correction was incorporated for all statistical test and significance was determined with a significance cutoff at 0.05. For beta diversity, the vegan R package was used to compute Bray Curtis dissimilarity matrix which was further analyzed with a permutational multivariate analysis of variance (PERMANOVA) test. Principal Coordinates Analysis (PCoA) plots were drawn based on Bray Curtis distance matrix. Comparisons of community composition were conducted at the ASV level.

To identify significant differences in taxonomic composition between visits or feeding groups, features with very low counts and those that are unlikely to be significant in comparison analyses were filtered out and excluded. This includes removal of features that were singletons and were only detected in a single sample. Additionally, features with variances that were among the lowest 10% were also removed to improve accuracy of comparative analyses. After subsequent filtering, 249 features which were composed of 72 genera, were used for subsequent analyses. Raw values of relative abundances are located in Additional File 2: Table S3. To assess taxonomic differences between groups and/or visits, multiple approaches were used. Analyses in R included Wilcoxon tests of taxonomic abundance, DESeq2 [95] and random forest classification.

Log2-fold change of median proportions were calculated, and significant testing was carried out. Pairwise Wilcoxon signed rank test with FDR correction was used to identify significantly different genera between visits for each feeding group and pairwise Wilcoxon rank sum test with FDR correction was used to compare genera between feeding groups at day 60. Metacoder [96] was used to generate heatmap trees. For visualization purposes, features that appeared in less than three samples or had fewer than 10 reads per sample and were not statistically significant were excluded from the heat trees (although still considered during significance testing). A reference tree was generated for the heatmap trees.

The random Forest package in R was used to carry out a random forest classification analysis to identify top predictors (genera) that discriminate between baseline and day 60 for each feeding group. DESeq2 was used to compare and identify specific ASVs that were significantly different between baseline and day 60 for each feeding group. ASVs with an FDR < 0.05 were considered significant. Shrinkage estimations of log2 fold change values were computed. A high positive fold-change does not necessarily reflect high abundance and should be interpreted as an increase in a specific ASV from baseline to Day 60.

## Supplementary information

**Additional file 1: Table S1.** Demographics of study participants. **Table S2**. Study protocol stool sample compliance criteria at Baseline, Day 30, and Day 60. **Figure S1**. Variable importance plots from random forest analysis using mean decrease in accuracy. **Figure S2**. Individual branched chain fatty acids; **a** isobutyrate and **b** isovalerate measured for the different feeding groups; amino acid (AAF; red), extensively hydrolyzed formula (EHF; green) and human milk (HM; blue). **Figure S3**. Spearman correlations of SCFA concentration with bacterial taxa. + indicates significant correlation and metabolite concentration (FDR < 0.05).

**Additional file 2: Table S3.** Relative abundances of amplicon sequence variants present in infant fecal samples throughout all treatment and timepoints. **Table S4.** Relative abundances of amplicon sequence variants present in individual infant fecal samples. Each column represents an indivdual fecal sample within a particular treatment and timepoint.

## Data Availability

The raw 16S rRNA sequences are deposited in the NCBI database under BioProject ID PRJNA612727 (https://www.ncbi.nlm.nih.gov/bioproject/612727).

## Abbreviations

EHF: Extensively hydrolyzed formula
AAF: Amino acid formula
HM: Human milk
16S rRNA: 16S ribosomal RNA
SCFA: Short chain fatty acids
BCFA: Branched chain fatty acids
ANOVA: Analysis of variance
CMA: Cow’s milk allergy
T_reg_ cells: Regulatory T cells
PCoA: Principal coordinate analysis
PERMANOVA: Permutational multivariate analysis of variance
ASV: Amplicon sequence variant
HMO: Human milk oligosaccharides
CHILD: Canadian healthy infant longitudinal development
GA: Gestational age
