# Supplementary material for: Stool microbiome, pH and short/branched chain fatty acids in infants receiving extensively hydrolyzed formula, amino acid formula, or human milk through two months of age
Source: BMC Microbiol. 2020 Nov 9;20:337. doi: 10.1186/s12866-020-01991-5 (PMC7650147; doi:10.1186/s12866-020-01991-5)
Supplement: Supplementary file 1 — Additional file 1: Table S1. Demographics of study participants. Table S2. Study protocol stool sample compliance criteria at Baseline, Day 30, and Day 60. Figure S1. Variable importance plots from random forest analysis using mean decrease in accuracy. Figure S2. Individual branched chain fatty acids; a isobutyrate and b isovalerate measured for the different feeding groups; amino acid (AAF; red), extensively hydrolyzed formula (EHF; green) and human milk (HM; blue). Figure S3. Spearman correlations of SCFA concentration with bacterial taxa. + indicates significant correlation and metabolite concentration (FDR < 0.05). [file 12866_2020_1991_MOESM1_ESM.docx]

TABLE S1. Demographics for study participants.

|  | Study Group, n (%) | | |  |
| --- | --- | --- | --- | --- |
|  | AAF | EHF | HM | *P* |
|  |  |  |  |  |
| Sex |  |  |  |  |
| Male | 11 (46) | 14 (54) | 10 (40) | 0.649 |
| Female | 13 (54) | 12 (46) | 15 (60) |  |
| Race |  |  |  |  |
| White | 20 (83) | 21 (81) | 24 (96) | 0.139 |
| Black | 0 (0) | 3 (12) | 0 (0) |  |
| More than one race | 4 (17) | 2 (8) | 1 (4) |  |
| Ethnicity |  |  |  |  |
| Hispanic | 1 (4) | 3 (12) | 3 (12) | 0.688 |
| Not Hispanic | 23 (96) | 23 (88) | 22 (88) |  |

TABLE S2 Study protocol stool sample compliance criteria at Baseline, Day 30, and Day 60

| Age (Days) | Study protocol compliance criteria |
| --- | --- |
| 1-7  (Baseline) | - meconium has passed - participant has not received study formula or has received study formula for ≤ 24 hours - no oral, intramuscular or intravenous antibiotic use by participant - no topical antibiotic use by participant in the diaper area 24 hours prior to stool collection - no probiotic use by participant - no HM feedings for formula-fed participants and no donor milk feedings in HM-fed participants |
| 30 | - no oral, intramuscular or intravenous antibiotic use by participant - no topical antibiotic use by participant in the diaper area 24 hours prior to stool collection - no probiotic use by participant - no more than two non-study formula feedings - no HM feedings for formula-fed participants, and no donor milk feedings in HM-fed participants |
| 60 |  |


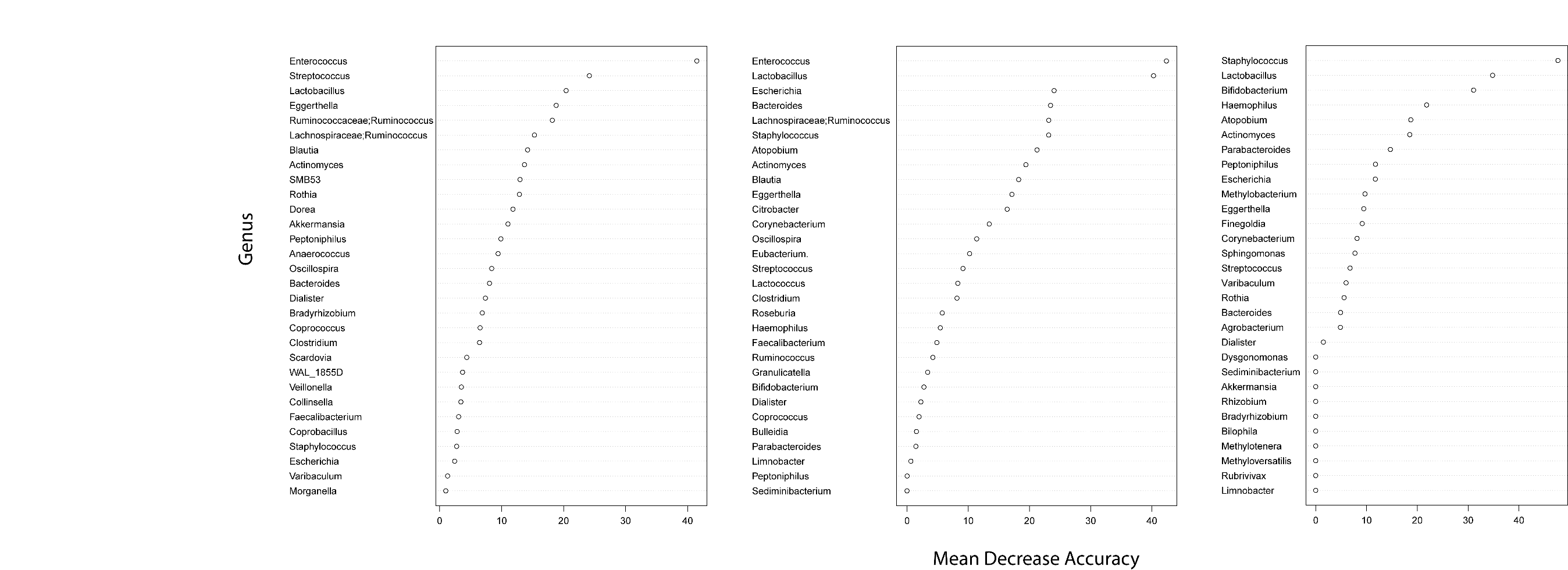


**b**

**c**

**a**

**Figure S1**. Variable importance plots from random forest analysis using mean decrease in accuracy. The top taxonomic (genus) predictors that differentiate between baseline and day 60 for each feeding group; **a** amino acid (AAF)**, b** extensively hydrolysed formula (EHF) and **c** human milk (HM) are presented here.


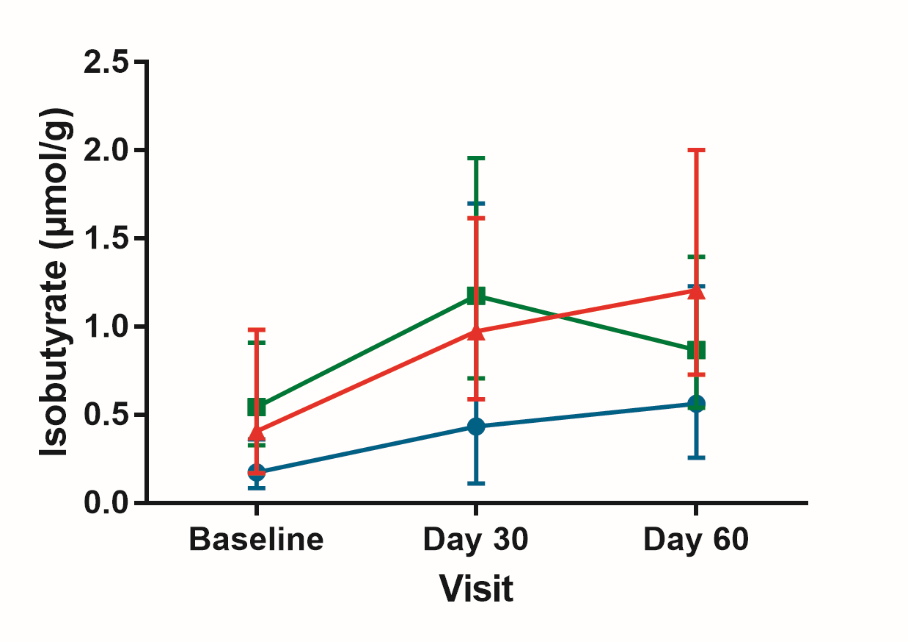

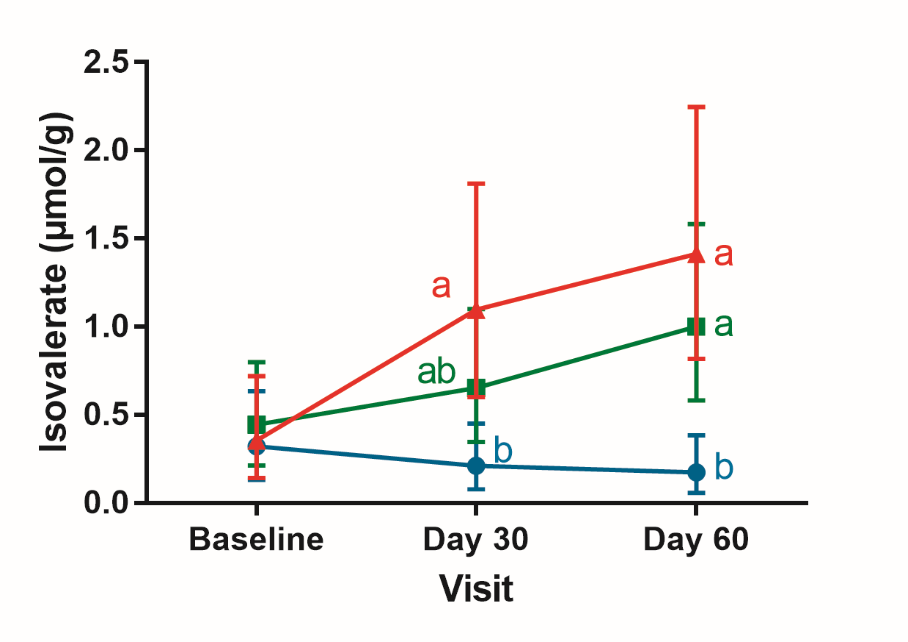


**a**

**b**

**Figure S2**. Individual branched chain fatty acids; **a** isobutyrate and **b** isovalerate measured for the different feeding groups; amino acid (AAF; red), extensively hydrolyzed formula (EHF; green) and human milk (HM; blue). To adjust for normality, a cubic transformation was carried out for isovalerate. For isobutyrate, an analysis of probability of a zero observation was carried out to account for its absence in most samples, followed by a modified log transformation.

**
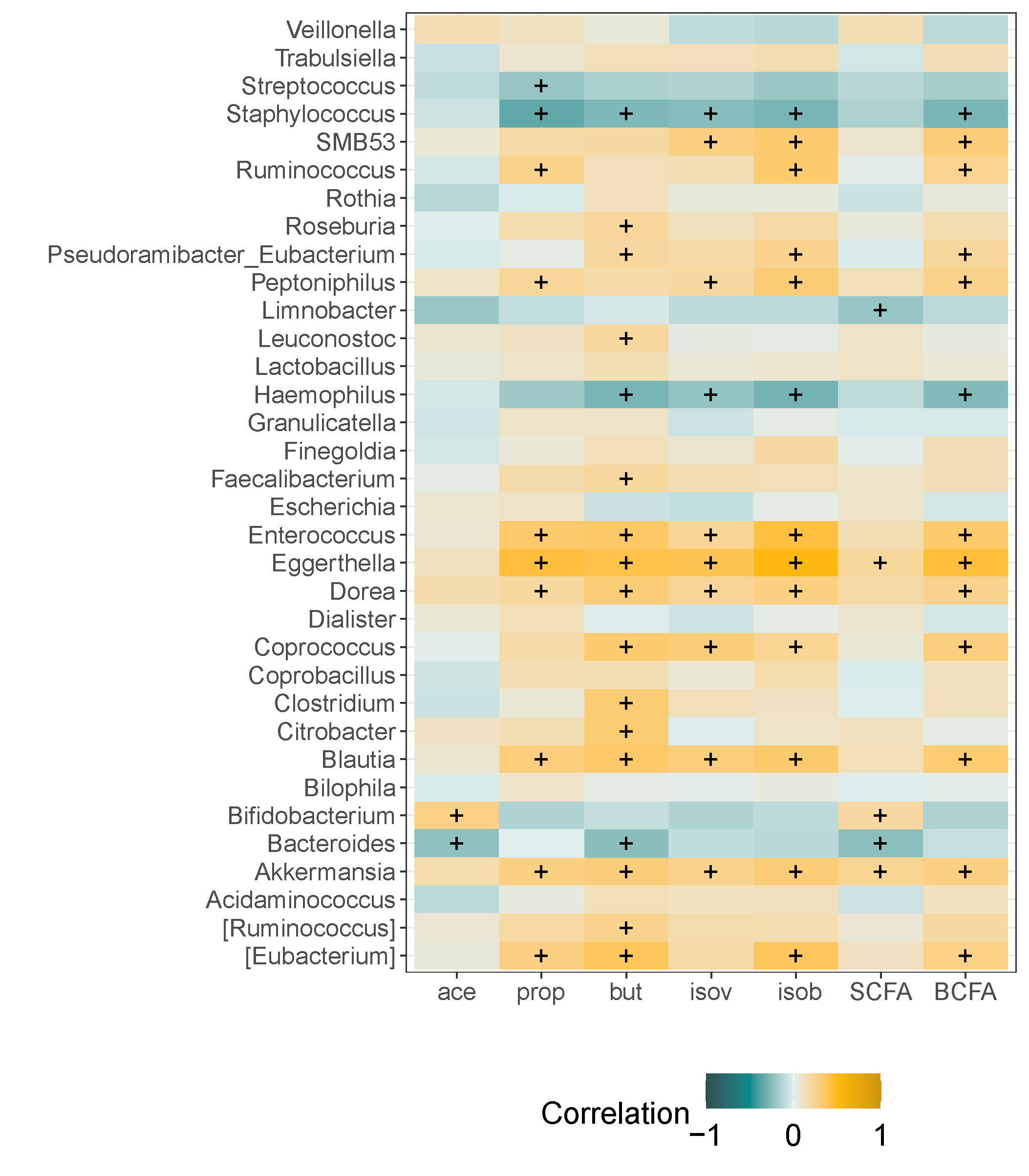
**

**Figure S3**. Spearman correlations of SCFA concentration with bacterial taxa. + indicates significant correlation and metabolite concentration (FDR<0.05). Only genera that had at least one significant correlation (FDR<0.05) with a metabolite are shown. Ace; acetate, prop; propionate, but; butyrate, isov; iso-valerate, isob: iso-butyrate, SCFA; total short chain fatty acid and BCFA; total branched chain fatty acids
